# Convergence effect during spatiotemporal succession of lacustrine plastisphere: loss of priority effects and turnover of microbial species

**DOI:** 10.1093/ismeco/ycae056

**Published:** 2024-04-18

**Authors:** Weihong Zhang, Shuxin Liang, Hans-Peter Grossart, Joseph Alexander Christie-Oleza, Geoffrey Michael Gadd, Yuyi Yang

**Affiliations:** Key Laboratory of Aquatic Botany and Watershed Ecology, Wuhan Botanical Garden, Chinese Academy of Sciences, Wuhan 430074, China; University of Chinese Academy of Sciences, Beijing 100049, China; Danjiangkou Wetland Ecosystem Field Scientific Observation and Research Station, Chinese Academy of Sciences & Hubei Province, Wuhan 430074, China; Key Laboratory of Aquatic Botany and Watershed Ecology, Wuhan Botanical Garden, Chinese Academy of Sciences, Wuhan 430074, China; School of Ecology and Environment, Tibet University, Lhasa 850000, China; Leibniz-Institute for Freshwater Ecology and Inland Fisheries (IGB), Neuglobsow 16775, Germany; Institute for Biochemistry and Biology, Potsdam University, Potsdam 14469, Germany; Department of Biology, University of the Balearic Islands, Palma 07122, Spain; Geomicrobiology Group, School of Life Sciences, University of Dundee, Dundee DD1 5EH, Scotland, United Kingdom; State Key Laboratory of Heavy Oil Processing, State Key Laboratory of Petroleum Pollution Control, China University of Petroleum, Beijing 102249, China; Key Laboratory of Aquatic Botany and Watershed Ecology, Wuhan Botanical Garden, Chinese Academy of Sciences, Wuhan 430074, China; University of Chinese Academy of Sciences, Beijing 100049, China; Danjiangkou Wetland Ecosystem Field Scientific Observation and Research Station, Chinese Academy of Sciences & Hubei Province, Wuhan 430074, China

**Keywords:** lacustrine plastisphere, priority effect, community turnover, community assembly, spatiotemporal succession

## Abstract

Succession is a fundamental aspect of ecological theory, but studies on temporal succession trajectories and ecological driving mechanisms of plastisphere microbial communities across diverse colonization environments remain scarce and poorly understood. To fill this knowledge gap, we assessed the primary colonizers, succession trajectories, assembly, and turnover mechanisms of plastisphere prokaryotes and eukaryotes from four freshwater lakes. Our results show that differences in microbial composition similarity, temporal turnover rate, and assembly processes in the plastisphere do not exclusively occur at the kingdom level (prokaryotes and eukaryotes), but also depend on environmental conditions and colonization time. Thereby, the time of plastisphere colonization has a stronger impact on community composition and assembly of prokaryotes than eukaryotes, whereas for environmental conditions, the opposite pattern holds true. Across all lakes, deterministic processes shaped the assembly of the prokaryotes, but stochastic processes influenced that of the eukaryotes. Yet, they share similar assembly processes throughout the temporal succession: species turnover over time causes the loss of any priority effect, which leads to a convergent succession of plastisphere microbial communities. The increase and loss of microbial diversity in different kingdoms during succession in the plastisphere potentially impact the stability of entire microbial communities and related biogeochemical cycles. Therefore, research needs to integrate temporal dynamics along with spatial turnovers of the plastisphere microbiome. Taking the heterogeneity of global lakes and the diversity of global climate patterns into account, we highlight the urgency to investigate the spatiotemporal succession mechanism of plastisphere prokaryotes and eukaryotes in more lakes around the world.

## Introduction

Microplastics are global pollutants, ubiquitous in a variety of environments around the world, which provide distinct habitats for microbial colonization from the surrounding environment, called the “plastisphere” [1, 2]. The plastisphere formed on microplastics represents an artificial environment and can span multiple biomes on Earth [1]. Furthermore, as a movable and durable vector in waterbodies, the plastisphere is constantly engaged in microbial exchange with the surrounding water, disturbing the native microbial communities with unknown knock-on effects on biogeochemical cycles and ecological risks of aquatic ecosystems [3–5]. Especially, the microbiota inhabiting this new artificial niche is becoming increasingly distinct from the surrounding habitat over time [6, 7]. Therefore, profiles of microbial colonization and succession in the plastisphere have recently attracted global interest. Unfortunately, little is known about the succession patterns and driving mechanisms for microbial communities during the continuous bilateral exchange between the plastisphere and the surrounding water.

Field studies have identified various microplastic polymers in natural aquatic ecosystems [8, 9]. Different microplastic polymers have different selective enrichment effects on microbial communities due to their differences in physiochemical characteristics (e.g. hydrophobicity and degradability) [10–12]. However, the surrounding environment seems to be more important than the polymer type in shaping the composition of bacterial communities in the plastisphere [13, 14]. Like in any biofilm, plastisphere formation comprises through microbial succession stages, a universal process to adapt to local environmental changes [15]. In particular, our previous study found that colonization time is the most important factor affecting the plastic microbial community in lakes [16]. However, to the best of our knowledge, in the plastisphere, microbial assembly and succession mechanisms both at the temporal and spatial scale are scarce, especially the assembly mechanism of microbial communities among different microplastic polymers and their succession mechanism on the spatiotemporal scales. This substantially limits our understanding of microbial succession and community dynamics on various microplastic types in natural aquatic ecosystems.

Ecosystem succession includes stochastic (e.g. priority effects) and, conversely, deterministic factors (e.g. competition and collaboration) [17]. Temporal succession of microbial communities in the plastisphere may depend on the cell’s order and/or timing of arrival, a phenomenon known as the “priority effect” [18, 19]. Temporal changes in spatial community patterns are mainly due to extinction (or local extinction) and settlement of species at different locations [20]. Arrival or disappearance of species may have feedback to local habitats, such as changes in niche availability for microbial life strategies of each new species and/or function recruited during community succession [15]. Therefore, extinction and colonization of microbial communities in the plastisphere need to be explicitly considered to measure their impact on microbial composition at the regional scale. However, little is known about the role and fate of primary colonizers in the plastisphere in relation to the temporal succession of subsequent communities. This substantially limits our understanding of microbial succession and community dynamics on various microplastic types in different natural aquatic ecosystems.

The assembly of microbial communities in the plastisphere follows basic processes related to diffusion, drift, competitive interactions, and environmental filtration [13, 14, 21, 22]. Although the mechanisms by which community structure is formed on microplastics have been thoroughly investigated [13, 14, 22–24], those that control ecological succession have remained elusive. Dynamics of species diversity on the two fundamental axes, i.e. space and time, are central to ecology [20, 25]. Spatial changes in species composition will increase over time, even under initially uniform environmental conditions, due to cumulative effects of diffusion, biological interactions, and ecological drift [20].

Therefore, the main objective of this study was to answer the following questions: (1) Does the plastisphere microbiome across various spatiotemporal conditions follow a similar succession pattern and converge or, by contrast, do these differentiate over time? (2) What is the role of priority effects and turnover in determining the composition and inheritance of the microbiomes in the plastisphere? (3) Are there any dynamic temporal patterns in the relative contribution of deterministic and stochastic processes for microbial community assembly in the plastisphere? We show that species turnover within microbial communities caused the loss of any priority effect, leading to a convergent succession of microbial communities in the plastisphere across various spatiotemporal conditions. Furthermore, it highlights that although prokaryotic and eukaryotic microbes in the plastisphere were often tightly entwined in the plastisphere, their colonization mechanisms and succession patterns varied at spatial and temporal scales. Finally, we demonstrate the dynamic behavior of stochastic and deterministic processes in controlling microbial community succession in the plastisphere.

## Materials and methods

### In situ field experiment and data collection

Nonbiodegradable (polypropylene, polyethylene], polyvinyl chloride, and polystyrene) as well as biodegradable (polylactic acid , polyhydroxyalkanoates], polybutylene succinate, and polybutylene adipate-co-terephthalate) polymer types were selected for microbial colonization in four freshwater lakes with different physicochemical properties and nutrient status (Table S1, Supplementary Materials). The field experiment was carried out from May to July 2021 in Wuhan, China. Briefly, 50 pellets (2–3 mm in diameter) of each polymer were incubated in metal canisters (diameter of 8.5 cm, height of 10.5 cm, and 0.8 mm pore size) as previously done in Yang *et al*. [23].

Ten pellets from each polymer type and each lake were collected after 3, 7, 15, 30, and 60 days of incubation Fig. S1). Additionally, at each time point, 2 L of water were collected from each lake. The water was processed by sequential filtration, i.e. a 156 μm prefiltration after which water was filtered through 2.0 μm (particle-associated, WP) and 0.22 μm (free-living, WF) pore-size membrane filters. Total DNA of the 160 microplastics and 40 water samples was collected using the CTAB method [26]. Extracted DNA was used as the template for Polymerase Chain Reaction amplification of the prokaryotic V3–V4 region of the 16S rRNA gene (using primers 341F (CCTAYGGGRBGCASCAG) /806R (GGACTACNNGGGTATCTAAT)) and the eukaryotic V4 region of the 18S rRNA gene (using primers 528F (GCGGTAATTCCAGCTCCAA) /706R (AATCCRAGAATTTCACCTCT)), both primer sets routinely used by Novogene Biotech (Beijing) with corresponding adaptors. Amplicon DNA libraries were sequenced on an Illumina Novaseq6000 platform (Illumina, San Diego, CA) at Novogene Biotech. Detailed sequencing procedures were provided as supporting information of Supplementary Materials and Methods. All raw sequence data of prokaryotic and eukaryotic communities are available in the Sequence Read Archive database (https://submit.ncbi.nlm.nih.gov/subs/sra/) under accession numbers PRJNA901233 and PRJNA901642, respectively.

### Statistical analysis

Permutational multivariate analysis of variance (PERMANOVA) was performed to assess the effects of colonization time, environment, and polymer types on the microbial composition of prokaryotic and eukaryotic communities in the plastisphere using the “vegan” R package [23]. To reveal spatiotemporal variations in the plastisphere prokaryotic and eukaryotic communities associated to eight different polymers, we utilized the “vegan” R package to calculate taxonomic similarity based on the 1-Bray–Curtis distance at the amplicon sequence variant (ASV) level, and the “ape” [27] and “picante” [28] R package to calculate the phylogenetic similarity based on the 1-βMNTD distance. Microbial community composition in the plastisphere was visualized using non-metric multidimensional scaling analysis (NMDS) based on Bray–Curtis dissimilarities. Then, the beta-diversity of the plastisphere prokaryotic and eukaryotic communities was partitioned into two components (species turnover and nestedness) using the “betapart” R package [29]. Specifically, β_ratio_ = β_sim_/β_SOR_, β_ratio_ > 0.5 indicates that beta diversity was determined dominantly by species turnover, and β_ratio_ < 0.5 indicates that nestedness was the dominant driver [30]. To reveal the rate of temporal turnover of the plastisphere prokaryotic and eukaryotic communities, the distance–decay relationship was investigated using the Sørensen similarity of microbial communities and change in Euclidean distance with colonization time [31]. The relative contributions of different ecological assembly processes of prokaryotic and eukaryotic communities were calculated using the Null model based on a framework and methods described previously [23, 32]. Detailed analysis methods of ecological assembly processes can be found in the Supplementary Materials and Methods.

## Results

### General overview of the lacustrine plastisphere

The lacustrine plastisphere exhibited dynamic multi-kingdom microbial communities over a period of 60 days. Colonization time and colonization environment were the two significant factors contributing to both of prokaryotic and eukaryotic community composition in the plastisphere, whereby spatiotemporal interactions also influenced the composition of the plastisphere microbiome (Table 1). Especially, colonization time, which nested on the colonization environment had the strongest influence on both prokaryotic and eukaryotic plastisphere microbiomes (Table S2). Consequently, we investigated the persistence of priority effects, species turnover patterns, and succession mechanisms of prokaryotic and eukaryotic plastisphere microbiomes in the four different freshwater lakes.

**Table 1:** PERMANOVA to assess the effect of colonization time, colonizing environment, and polymer type on the composition of prokaryotic and eukaryotic microbial communities in the plastisphere.

Factors	Prokaryotic microbiome		Eukaryotic microbiome	
	R^2^	*P*-value	R^2^	*P*-value
Colonization time	0.2682	0.0001^*^^*^^*^	0.1917	0.0001^*^^*^^*^
Colonizing environment	0.0731	0.0001^*^^*^^*^	0.0711	0.0001^*^^*^^*^
Polymer type	0.0760	0.0001^*^^*^^*^	0.0308	1.0000
Colonization time×Colonizing environment	0.1586	0.0001^*^^*^^*^	0.2124	0.0001^*^^*^^*^
Colonization time×Polymer type	0.1191	0.7956	0.1031	1.0000
Colonizing environment×Polymer type	0.0699	1.0000	0.0815	1.0000
Residuals	0.2352		0.3094	

Asterisks indicate statistical significance (^*^^*^^*^P-value < 0.001). “×” indicates the interaction.

### Microbial composition varies in the plastisphere of different lakes

PERMANOVA results showed that the composition of microbial communities (both prokaryotic and eukaryotic communities) was different among the four different lakes (whether WP, Table S3), indicating that these four different lakes provide different sources of microbes to “seed” the plastisphere. However, the composition of plastisphere clearly differed from that of the WP and the WF (Fig. S2). Besides, the composition of microbial communities, including prokaryotes and eukaryotes, within the plastisphere significantly differed across the studied lakes (Figs S3 and S4). For instance, on the third day of microbial colonization, prokaryotes in the plastisphere of Lake 1 were predominantly comprised of *Proteobacteria* (88.07%) and *Actinobacteria* (1.98%), while *Proteobacteria* (79.37%) and *Cyanobacteria* (7.55%) constituted the main components of prokaryotes in the plastisphere of Lake 2 (Fig. S3). Specifically, *Proteobacteria* in the plastisphere were mainly composed of *Gammaproteobacteria* and *Alphaproteobacteria* (Fig. S4). Similarly, at the same colonization stage, eukaryotic communities in the plastisphere of Lake 1 were mainly composed of *Ciliophora* (32.12%) and *Ascomycota* (24.47%), whereas *Ciliophora* (20.70%) and *Rotifera* (12.90%) dominated the eukaryotes in the plastisphere of Lake 2 (Fig. S3). These findings indicate that the composition of early colonizers of both prokaryotic and eukaryotic communities within the plastisphere varies across lakes with different environmental characteristics (Fig. S5). Furthermore, even in the same lake, the community composition of plastisphere microbiomes differed between different successional stages (Fig. S6). For example, in contrast to Day 3 of the incubation, on Day 60, plastisphere prokaryotic communities in Lake 1 were mainly composed of *Proteobacteria* (80.29%) and *Bacteroidota* (6.14%), and the plastisphere eukaryotes were mainly composed of *Ciliophora* (37.07%) and *Arthropoda* (24.95%) (Fig. S3).

In general, the influence of colonization time nested in the colonized environment on microbial community composition in the plastisphere was greater for prokaryotes than for eukaryotes (Table S2). On the contrary, the influence of the colonized environment nested in the colonization time on microbial composition in the plastisphere was greater for eukaryotes than prokaryotes (Table S2). This indicates that plastisphere prokaryotes are more susceptible to colonization time at the same colonized environment, i.e. succession, while plastisphere eukaryotes were more susceptible to environmental factors at the same colonization time.

### Response of plastisphere microbial communities to colonization time and colonized environment

**Figure 1:**
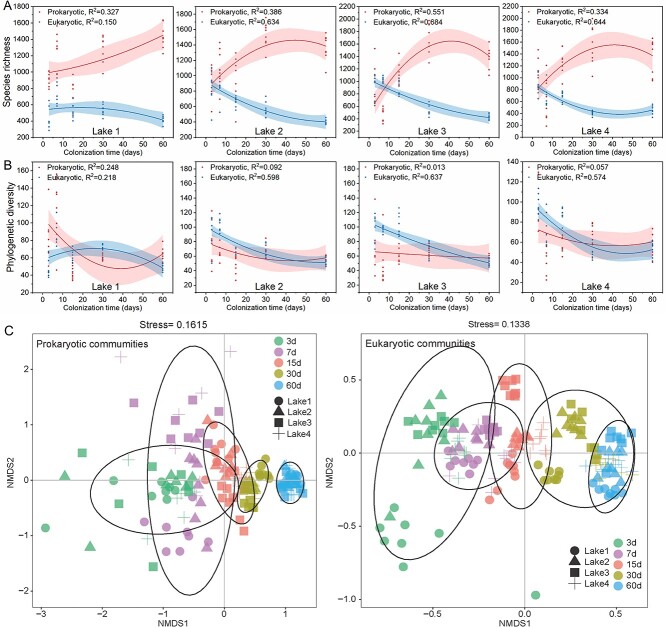
Responses of species richness (A) and phylogenetic diversity (B) of plastisphere microbial communities in four different lakes to colonization time; (C) NMDS results showing that the differences of the plastisphere microbial communities between the four different lakes also showed a convergence effect, especially the prokaryotic communities.

In Lake 1, species richness of the plastisphere prokaryotes increased with colonization time, while in all other lakes, it initially increased and then decreased (Fig. 1A). This indicates that the response of prokaryotic species richness to colonization time was different in different lakes during the plastisphere succession. Meanwhile, responses of phylogenetic diversity of plastisphere prokaryotes to colonization time differed from responses of their species richness to colonization time (Fig. 1B). For example, the phylogenetic diversity of plastisphere prokaryotes in Lake 1 initially decreased and then slightly increased with colonization time, while it kept relatively stable in all other lakes. For plastisphere eukaryotes, species richness and phylogenetic diversity responses to colonization time differed from plastisphere prokaryotes. In Lake 1, initially, species richness of plastisphere eukaryotes increased slightly and then decreased over time, while in Lakes 2 and 3 it decreased, and in Lake 4 it first decreased and then increased (Fig. 1A). However, phylogenetic diversity of plastisphere eukaryotes was similar to response patterns of species richness to colonization time (Fig. 1B). This may imply that plastisphere eukaryotes were more consistent in simultaneous succession of taxonomic and phylogenetic composition than plastisphere prokaryotes.

Effects of colonization time on composition of plastisphere microbial communities in different lakes through NMDS analysis (Fig. S6) revealed that in the same lake differences between microbial communities of different microplastic polymers decreased over time, yielding a convergence effect. Moreover, plastisphere microbial communities among all four lakes showed a convergence effect, especially prokaryotic communities (Fig. 1C).

### Environmental factors that influence plastisphere microbial communities

**Figure 2:**
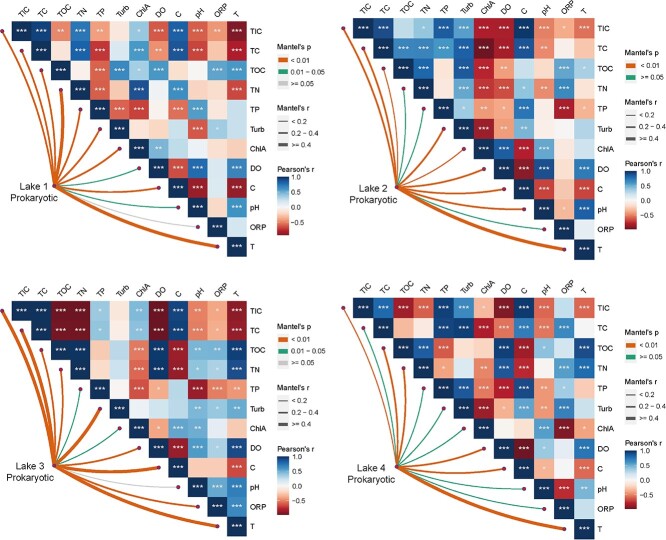
Mantle test to elucidate the response of prokaryotic community plastisphere composition to environmental factors in the surrounding water of all lakes; TIC, total inorganic carbon; TC, total carbon; TOC, total organic carbon; TN, total nitrogen; TP, total phosphorus; Turb, turbidity; ChlA, chlorophyll a content; DO, dissolved oxygen; C, electrical conductivity; T, temperature.

Mantel test showed that the composition of plastisphere prokaryotic communities from different lakes was shaped by different environmental factors in the surrounding water (Fig. 2). In Lake 1, community composition of plastisphere prokaryotes was mainly affected by total nitrogen (TN, r = 0.58, *P*-value < .01) and temperature (T, r = 0.47, *P*-value < 0.01), while composition of the plastisphere prokaryotic communities in Lake 2 and 4 was mainly affected by T (r = 0.44, *P*-value < 0.01 and r = 0.49, *P*-value < 0.01, respectively). Composition of the plastisphere prokaryotic communities in Lake 3 was mainly affected by turbidity (Turb, r = 0.50, *P*-value < 0.01), electrical conductivity (C, r = 0.47, *P*-value < 0.01), and T (r = 0.41, *P*-value < 0.01). Similarly, the community composition of plastisphere eukaryotes was shaped by varying environmental factors in the surrounding water (Fig. S7). In Lake 1, community composition of plastisphere eukaryotes was mainly affected by TN (r = 0.59, *P*-value < 0.01), total carbon (TC, r = 0.56, *P*-value < 0.01), and total phosphorus (TP, r = 0.52, *P*-value < 0.01), while composition of plastisphere eukaryotic communities in Lake 2 was mainly affected by Turb (r = 0.46, *P*-value < .01), T (r = 0.42, *P*-value < 0.01), and total organic carbon (TOC, r = 0.40, *P*-value < 0.01). Composition of plastisphere eukaryotic communities in Lake 3 was mainly affected by Turb (r = 0.54, *P*-value < 0.01), T (r = 0.48, *P*-value < 0.01), and oxidation-reduction potential (r = 0.44, *P*-value < 0.01), while composition of plastisphere eukaryotic communities in Lake 4 was mainly affected by T (r = 0.50, *P*-value < .01) and chlorophyll a ( r = 0.44, *P*-value < 0.01). These results also indicate that plastisphere prokaryotic and eukaryotic communities are also affected by differencing environmental factors in the same lake.

### Community variation patterns of plastisphere microbial communities from different colonized environments

**Figure 3:**
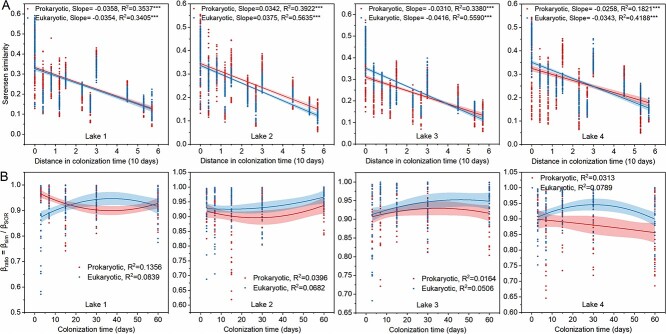
Composition variation patterns of plastisphere prokaryotic and eukaryotic communities from different lakes; (A) time decay in the similarity of the plastisphere prokaryotic and eukaryotic communities indicating rates of community turnover; asterisks indicate statistical significance (^*^^*^^*^P-value < .001); (B) variation patterns of the plastisphere prokaryotic and eukaryotic community composition on different polymers at different succession stages; β_ratio_ = β_sim_/β_SOR_. β_ratio_ > 0.5 indicates that beta diversity was determined dominantly by species turnover, and β_ratio_ < 0.5 indicates nestedness was the dominant driver.

The β_ratio_ of the plastisphere prokaryotic and eukaryotic communities in different lakes during the entire succession process was more than 0.5 (Fig. S8), indicating that variations in the composition of plastisphere microbial communities were dominated by species turnover. There was also a difference in rates of temporal turnover between the plastisphere prokaryotic and eukaryotic communities as well as different environmental factors. Plastisphere prokaryotic communities in Lake 1 showed a strong temporal pattern (R^2^=0.3537), which exhibited the fastest temporal turnover (Slope=0.0358) (Fig. 3A). Plastisphere eukaryotic communities in all other lakes also showed a strong temporal pattern, which exhibited a faster temporal turnover than of the prokaryotic communities in the plastisphere. Furthermore, the rate of species turnover of plastisphere microbial communities in different lakes differed greatly. Temporal turnover rate of the plastisphere prokaryotic communities in Lake 1 was ~1.39 times faster than in Lake 4, while temporal turnover rate of plastisphere eukaryotic communities in Lake 3 was roughly 1.21 times faster than in Lake 4 (Fig. 3A).

In the same lake, the contribution of species turnover to variations in the composition of plastisphere microbial communities at different colonization stages also differed (Fig. 3B). The β_ratio_ of plastisphere prokaryotic communities in Lakes 1 and 2 first decreased and then increased, while in Lake 3 it first increased and then decreased. Furthermore, the β_ratio_ of the plastisphere prokaryotic communities in Lake 4 decreased with increasing colonization time, while the β_ratio_ of plastisphere eukaryotic communities first increased and then decreased. This shows that the response of the β_ratio_ of plastisphere eukaryotic communities to colonization time differed from prokaryotic communities.

### Assembly processes and response to colonization time of plastisphere microbial communities in contrasting lakes

**Figure 4:**
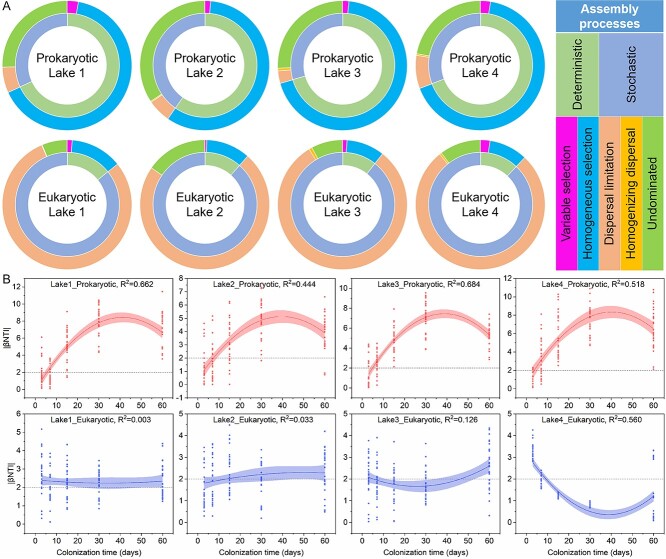
Ecological assembly processes of prokaryotic and eukaryotic plastisphere communities in four contrasting lakes and their response to colonization time; (A) relative contributions of different ecological processes to the assembly of prokaryotic and eukaryotic plastisphere communities in all lakes; (B) stochastic and deterministic responses to colonization time during plastisphere microbial succession; |βNTI| > 2 represents deterministic processes; |βNTI| < 2 represents stochastic processes.

In four contrasting lakes, microbial communities revealed similar assembly processes during the plastisphere colonization succession, while prokaryotic and eukaryotic plastisphere microbiomes from the same lake showed different assembly processes (Fig. 4A). Assembly of the prokaryotic plastisphere microbiome was dominated by deterministic processes, while assembly of the plastisphere eukaryotic microbiome was dominated by stochastic processes. Specifically, the assembly of prokaryotic plastisphere communities was driven by homogeneous selection, while dispersal limitation dominated the assembly of plastisphere eukaryotic communities. Homogenizing processes drove the assembly of plastisphere prokaryotic communities, while differentiating processes dominated the assembly of plastisphere eukaryotic communities (Fig. S9).

With increasing colonization time, the assembly of prokaryotic communities in different lakes changed from stochastic to deterministic processes (Fig. 4B). However, the response of ecological processes in eukaryotic community assembly in different lakes to colonization time also differed. Assembly of plastisphere eukaryotic communities in Lakes 1, 3, and 4 was dominated by deterministic processes on the third day of incubation. However, the relative contribution of deterministic processes to the assembly of plastisphere eukaryotic communities in Lake 1 revealed some slight fluctuation with increasing colonization time. The relative contribution of deterministic processes to the assembly of plastisphere eukaryotic communities in Lakes 3 and 4 first declined and then increased with colonization time. In Lake 2, the assembly of plastisphere eukaryotic communities even changed from stochastic to deterministic processes with colonization time. In general, the relative contribution of deterministic processes to plastisphere eukaryotic communities increased with colonization time.

## Discussion

Succession forms the basis of ecological theory; yet, few studies have sought generalizations for a range of habitats, especially temporal succession of microbial communities in the plastisphere of contrasting freshwater lakes. Such comparative studies are necessary because they provide insight into community assembly mechanisms (e.g. environmental filtering, biological interactions, priority effects, and diffusion constraints) at different successional stages and spatial or temporal scales [33]. Lakes are an important ecosystem where microbial communities gather, and these microorganisms provide an important microbial source for the formation of the plastisphere on sterile microplastics [23]. Especially, the geographic isolation of relatively enclosed freshwater lakes provides ideal habitats to explore environmental effects on microbial successions in the plastisphere. We found obvious differences in the composition of free-living and particle-associated microbial communities between the four studied lakes. Such findings provide a foundation for the exploration of the temporal succession mechanisms of the plastisphere microbial communities in the different lakes because the different microbiomes compositions of these lakes constitute different sources for microbial colonization of the microplastics. In this research, the microorganisms in the surrounding water continuously colonized the microplastics and formed a dynamic multi-kingdom community in the plastisphere during 60 days of incubation.

### Succession of microbial communities in the plastisphere represents a process of continuous invasion

Sterile microplastics provide hotspots for colonization for both free-living and particle-associated microbial communities when entering lake ecosystems [21]. However, sterile microplastic surfaces are often difficult for microbes to colonize, e.g. due to a lack of readily available nutrients or the presence of toxic compounds [34–36]. Thus, pioneer species may actively change their environment (i.e. biofilm formation) to facilitate the invasion of new species that would otherwise not survive and/or thrive [30]. This study showed that *Alphaproteobacteria* and *Gammaproteobacteria* were the main prokaryotic colonizer species in the early plastisphere, which have also been recognized as pioneer colonizers of the plastisphere in aquatic environments [37, 38]. Besides, we found that the microbial pioneer colonizers of the plastisphere also include primary producers such as *Diatoms*, *Chlorophyta*, and *Cyanobacteria*. These microorganisms adhere to microplastic surfaces through pilli, protein adhesion, the production of complex extracellular polymeric substances, and stabilizing intercellular interactions [39]. Microplastics are in direct contact with the surrounding water during microbial succession in the plastisphere, i.e. changes in microbial communities colonizing the surface of microplastics are continuously exchanged with microorganisms in the surrounding water [13, 40]. However, as the succession of microbial communities in the plastisphere progresses, resident taxa may occupy more ecological niches and monopolize resource availability in colonized environments, thereby reducing further microbial invasions [41].

### The loss of primary colonizers leads to a convergent succession of microbial communities in the plastisphere

In all of our freshwater lakes, the community composition varied greatly during the succession of plastisphere microbial communities, which were mainly affected by species turnover. Species turnover represents the substitution of species over time, whereby some species are replaced by others within environmental filtration and spatial and/or historical constraints [42]. In addition, variations in microbial communities on different microplastic polymers in the same lake were also dominated by species turnover, presumably because of different selection and enrichment of microbial communities by the different microplastic types [21, 43, 44].

Invasive species may benefit from priority effects more than plastisphere priority colonizers, amplifying the spatiotemporal distribution and ecological impact of species establishing later in the plastisphere [45]. Particularly, random fluctuations and genetic variation (drift and diversification) are inherent properties of time-dependent ecological studies, leading to divergent or convergent communities [46]. Composition of such microbial pioneer species varies among different microplastics under the same environmental background due to the selective enrichment of microbial communities on different microplastic types [40, 47, 48]. These microorganisms form early microplastic biofilms to resist the unfavorable environment on fresh microplastics enabling the formation of early microbial colonizer communities [1, 39]. These early biofilms provide a good basis for the development of more complex microbial communities at later colonization stages, which seem to reduce the impact of different microplastic polymers on the plastisphere microbial communities. This also explains why the succession of microbial communities on different microplastic types in the same lake is increasingly convergent over time.

Moreover, we found that plastisphere microbial communities in all lakes were impacted differently by various environmental factors. This may be the reason for the different turnover rates of microbial community in different lakes. Similarly, even in the same lake, the environmental influences of the prokaryotic and eukaryotic communities are different, which may be an important reason for the different turnover rates of the prokaryotes and eukaryotes in the plastisphere. However, plastisphere microbial communities in different lakes also showed a convergent development, especially prokaryotes. One of the reasons may be that the effect of polymer types on the plastisphere prokaryotic communities was greater than that on eukaryotic communities. Another reason could be that the influence of spatiotemporal factors may more efficiently mask the selective effects of polymer types on eukaryotic communities in the plastisphere [4]. Thus, our results suggest that although environmental factors in the surrounding water and biofilm formation on the microplastics were dominant forces driving local plastisphere microbial communities, community composition in the plastisphere may still be influenced by polymer types, but gradually converge during biofilm development [11].

### Variable priority effects of plastisphere microbial communities lead to different succession trajectories in contrasting lakes

There is growing evidence that the first colonizers play an important role in the success of microorganisms establishing and colonizing plastic surfaces [1, 37, 49]. Especially, the priority effect will affect subsequent community succession and pose a long-term impact on microbial community composition [45]. Our study revealed that microbial communities forming the early plastisphere in different lakes varied due to differences in the composition of microbial communities in the surrounding water. Earlier, it has been reported that priority effects may affect the success of invasive species [45]. Differences in priority colonizers on plastics may lead to variations in succession trajectories. Therefore, we analyzed the time series of species richness and phylogenetic diversity of prokaryotic and eukaryotic plastisphere communities in various lakes. The obtained results show that succession trajectories of plastisphere microbial communities were distinct for each lake. The reason may be that the spatiotemporal succession of each plastisphere microbial community follows different trajectories depending on the initial environment and microbial colonizers of each lake.

To date, several studies have measured spatiotemporal succession dynamics of microbial communities and proposed certain principles to guide bacterial coexistence, including deterministic processes based on niche theory and stochastic processes based on neutral theory [50, 51]. However, the assembly mechanisms expressed during the succession of plastisphere microbial communities remain poorly understood. Particularly, the relative contribution of stochastic and deterministic processes controlling the distribution patterns during the succession of prokaryotic and eukaryotic communities in the plastisphere remains unknown. Here, we show that the assembly of the plastisphere prokaryotic community at the early stage is mainly a stochastic process, but increasingly changes to a deterministic process during successional progression. Although plastisphere prokaryotic communities in different lakes show consistent changes in assembly mechanisms with colonization time, the plastisphere eukaryotic communities do not reveal any consistent changes in assembly mechanisms. From this, we conclude that the influence of colonization time on plastisphere prokaryotic community assembly was greater than on eukaryotes in the same environment, while the influence of environmental factors on assembly of the plastisphere eukaryotic communities was stronger than on prokaryotes at the same colonization time point.

### Plastisphere microbial communities in different lakes had similar assembly processes across the whole plastisphere succession

During the whole succession process, the plastisphere prokaryotic communities of different lakes had similar ecological assembly processes, and so did the eukaryotes. However, assembly mechanisms of the plastisphere prokaryotic and eukaryotic communities in the same lake were distinct during the whole succession process. Temporal variations in spatial community changes are mainly due to local extinctions and species settlement [52]. Therefore, we need to know how species loss or gain results in differences in species richness between different communities as species richness differences are known to be caused by species loss and gain [23]. In our study, we found that species richness of the plastisphere prokaryotic communities increased with colonization time, while the plastisphere eukaryotic communities decreased. In natural communities, usually, two processes (extinction and colonization) and two distinct outcomes (homogenization and heterogeneity) occur simultaneously [20]. When rare or infrequent species become extinct within a region, i.e. extinction results in homogenizing processes, but it can also lead to differentiating processes [20]. Results of the Null model showed that the assembly of the plastisphere prokaryotic communities was dominated by homogenizing processes, while assembly of eukaryotic communities was dominated by differentiating processes. Based on these results, we conclude that homogenization of the plastisphere prokaryotic communities occurs because the rate of species increase was higher than that of extinction. In contrast, the plastisphere eukaryotic communities become more heterogeneous when the rate of increase is lower than that of extinction. This may be an important reason for the observed differences in assembly processes of the plastisphere prokaryotic versus eukaryotic communities during spatiotemporal succession in different lakes. In addition, the plastisphere eukaryotic communities are affected by stronger dispersal limitation than prokaryotes, which may lead to increased niche differentiation. This could be another important reason for the observed differences in assembly processes between prokaryotic and eukaryotic communities in the plastisphere.

**Figure 5:**
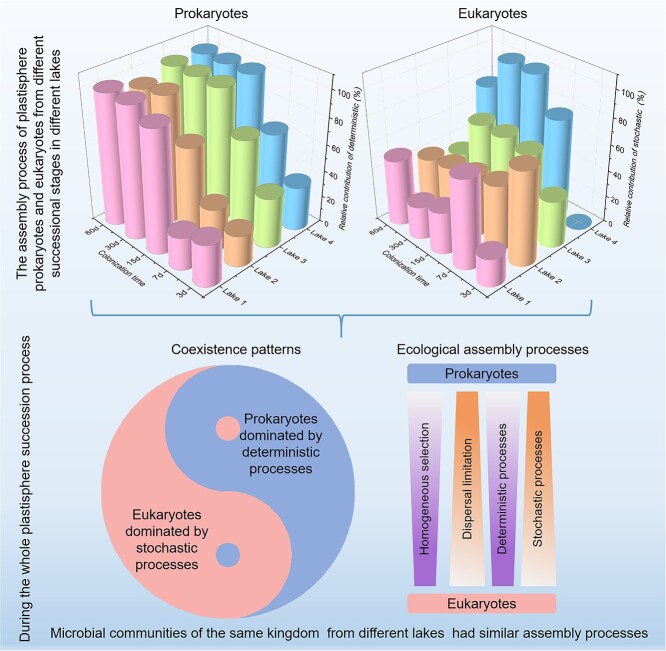
Conceptual model revealing assembly mechanisms of microbial plastisphere communities during spatiotemporal succession.

Overall, we conceptualized assembly mechanisms of the plastisphere microbial communities on different polymers during their spatiotemporal succession (Fig. 5). In different lakes, distinct assembly patterns of the plastisphere microbial communities depend on colonization time and colonizers within different kingdoms, while microbial communities of the same kingdom in the plastisphere had similar assembly processes during the 60-day succession. This is like the yin-yang balance in the Tai Ji, i.e. the assembly of the plastisphere prokaryotes was dominated by deterministic processes, while the assembly of the plastisphere eukaryotes was dominated by stochastic processes.

## Conclusion and implications

Our results clearly demonstrated that the spatiotemporal assembly of plastisphere microbiomes mainly depends on colonization time and colonizers within different kingdoms, and the plastisphere prokaryotes had stronger time temporal patterns in community composition and assembly than eukaryotes. These results expand our understanding of the temporal succession and ecological processes behind changes in the plastisphere microbial communities at the kingdom level under changing environments and underscore the implications of predicted plastisphere ecological consequences. The gain and loss of microbial diversity in different kingdoms during succession in the plastisphere potentially impact the stability of entire microbial communities and related biogeochemical cycles. Therefore, future research on the plastisphere microbiome needs to integrate the temporal dynamics along with their spatial turnovers. Especially, considering the heterogeneity of global lakes and the diversity of global climate, we highlight the urgency to investigate the spatiotemporal succession mechanism of plastisphere prokaryotes and eukaryotes in more lakes around the world.

## Author contributions

Weihong Zhang and Yuyi Yang (Conceptualization); Weihong Zhang and Shuxin Liang (Methodology); Weihong Zhang (Writing—original draft); Weihong Zhang (Visualization); Hans-Peter Grossart, Joseph Alexander Christie-Oleza, Geoffrey Michael Gadd, and Yuyi Yang (Reviewing, validation, and editing).

## Conflicts of interest

The authors declare no conflict of interest.

## Funding

This work was supported by the National Natural Science Foundation of China [Grant No. 32071614], the Starting Research Fund and Opening Research Fund from Key Laboratory of Aquatic Botany and Watershed Ecology, Chinese Academy of Sciences [Grant No. Y9519802 and E0520202], and the Science and Technology Development Plan Project of Hangzhou [Grant No. 202204T14]. H.P.G. was supported by the European Union’s Horizon 2020 Research and Innovation programme, under the Grant Agreement number 965367 (PlasticsFatE).

## Data availability

The raw prokaryotic and eukaryotic community gene sequences generated in the present study were deposited in the Sequence Read Archive (SRA) database (https://submit.ncbi.nlm.nih.gov/subs/sra/) under accession number PRJNA901233 and PRJNA901642.

## Supplementary Material

Supplementary_materials_R2_clean_ycae056
