# Supplementary material for: Convergence effect during spatiotemporal succession of lacustrine plastisphere: loss of priority effects and turnover of microbial species
Source: ISME Commun. 2024 Apr 18;4(1):ycae056. doi: 10.1093/ismeco/ycae056 (PMC11073396; doi:10.1093/ismeco/ycae056)
Supplement: Supplementary_materials_R2_clean_ycae056 [file supplementary_materials_r2_clean_ycae056.docx]

**Supplementary materials**

**Convergence effect during spatiotemporal succession of lacustrine plastisphere: loss of priority effects and turnover of microbial species**

Weihong Zhang^1,2,3^, Shuxin Liang^1,4^, Hans-Peter Grossart^5,6^, Joseph Alexander Christie-Oleza^7^, Geoffrey Michael Gadd^8,9^, Yuyi Yang^1,2,3,*^

^1^ Key Laboratory of Aquatic Botany and Watershed Ecology, Wuhan Botanical Garden, Chinese Academy of Sciences, Wuhan, 430074, China

^2^ University of Chinese Academy of Sciences, Beijing, 100049, China

^3^ Danjiangkou Wetland Ecosystem Field Scientific Observation and Research Station, Chinese Academy of Sciences & Hubei Province, Wuhan, 430074, China

^4^ School of Ecology and Environment, Tibet University, Lhasa 850000, China

^5^ Leibniz-Institute for Freshwater Ecology and Inland Fisheries (IGB), Neuglobsow 16775, Germany.

^6^ Institute for Biochemistry and Biology, Potsdam University, Potsdam 14469, Germany.

^7^ Department of Biology, University of the Balearic Islands, Palma 07122, Spain.

^8^ Geomicrobiology Group, School of Life Sciences, University of Dundee, Dundee DD1 5EH, Scotland, UK.

^9^ State Key Laboratory of Heavy Oil Processing, State Key Laboratory of Petroleum Pollution Control, China University of Petroleum, Beijing 102249, China.

***Corresponding author**

E-mail: yangyy@wbgcas.cn (Yuyi Yang)

Address: Wuhan Botanical Garden, Lumo Road No.1, Wuchang District, Wuhan, P.R. China. Phone: +86-27-87700853; Fax: +86-27-87510251.

**Supplementary materials and methods**

**In-situ field experiments**

Microplastic samples were white spheres with a particle size of 2-3 mm diameter, purchased from Guangdong Tesulang Chemical, China. All the microplastics were ultrasonically precleaned with 75% ethanol for 30 min and washed several times with sterile ultrapure water to eliminate any associated microorganisms before the experiments. 50 grains of the sterilized microplastics were then loaded into a cylindrical stainless steel cage (diameter 8.5 cm, height 10.5 cm, and pore size approximately 0.8 mm) and placed 0.5 m below the surface of the water in the four lakes of differing nutrient status [1].

Microplastic samples in each lake were collected at 3, 7, 15, 30, and 60 days after placement. Two fractions of water samples were obtained by sequential 156 μm prefiltering water through 2.0 μm (particle-associated, WP) and 0.22 μm (free-living, WF) pore-size membrane filters. Finally, a total of 160 microplastic samples and 40 water samples were collected. All microplastic samples and filtered membranes were immediately stored at −80 °C before DNA extraction.

**Sequencing of prokaryotic and eukaryotic communities**

The total DNA of each sample was extracted according to the CTAB method [2]. 16S rRNA gene V3-V4 barcoded primers 341F/806R and the 18S rRNA gene V4 barcoded primers 528F /706R were amplified and purified in each sample to investigate the composition of the prokaryotic and eukaryotic communities [3], respectively. Qualified DNA libraries were sequenced by the Illumina Novaseq6000 platform (Illumina, San Diege, CA) at Novogene Biotech (Beijing). The raw data obtained was quality controlled using fastp software, and then the Usearch software was used to detect and remove chimeras, resulting in final effective data. The DADA2 module in QIIME2 software (QIIME2-2020.6) was used for noise reduction to obtain the final amplicon sequence variant (ASV). The resulting ASVs were then compared with the SILVA database using the classify-sklearn module in QIIME2 software to obtain species information for each ASV.

***Quantitative analysis of ecological assembly processes of microbial communities in the plastisphere***

The null model was applied to calculate the relative contributions of ecological assembly processes of plastisphere prokaryotic and eukaryotic communities using the framework described by Stegen et al., 2012 [4]. Briefly, |βNTI| > 2 indicates the deterministic processes, while the |βNTI| < -2 indicates the stochastic processes. Besides, βNTI < -2 indicates the homogenous selection, while βNTI >2 indicates the variable selection. Moreover, the |βNTI| < 2 and RC_Bray_ < -0.95 indicates the homogenizing dispersal, |βNTI| < 2 and RC_Bray_ >0.95 indicates the dispersal limitation, and the |βNTI| < 2 and |RC_Bray_| < 0.95 indicates the “undominated” processes, which mostly consists of weak selection, weak dispersal, diversification, and/or drift. Homogenising = Homogeneous selection + Homogenising dispersal; Differentiating = Variable selection + Dispersal limitation [5, 6].

**Table S1**. Information on the geography of the test site, physicochemical properties, and nutrient status of water in the lake ecosystems during the spatiotemporal succession of the plastisphere.

|  | L1-3d | L1-7d | L1-15d | L1-30d | L1-60d | L2-3d | L2-7d | L2-15d | L2-30d | L2-60d |
| --- | --- | --- | --- | --- | --- | --- | --- | --- | --- | --- |
| Latitude | 30.546 | 30.546 | 30.546 | 30.546 | 30.546 | 30.544 | 30.544 | 30.544 | 30.544 | 30.544 |
| Longitude | 114.427 | 114.427 | 114.427 | 114.427 | 114.427 | 114.424 | 114.424 | 114.424 | 114.424 | 114.424 |
| T | 25.2 | 23.8 | 26.4 | 27 | 30.4 | 24.2 | 23.1 | 25.3 | 25.5 | 29.8 |
| DO | 1.58 | 1.40 | 1.30 | 1.23 | 2.03 | 1.27 | 1.16 | 1.09 | 0.73 | 1.91 |
| C | 391.5 | 369.5 | 322.5 | 361.1 | 260.4 | 353.6 | 363.1 | 405.6 | 390.1 | 290.3 |
| pH | 8.32 | 8.48 | 8.46 | 8.28 | 8.70 | 7.72 | 7.81 | 7.98 | 7.64 | 8.33 |
| ORP | 76.9 | 77.1 | 49.6 | 95.1 | 81.7 | 88.7 | 93.0 | 61.2 | 112.5 | 93.6 |
| Turb | 43.3 | 34.4 | 39.87 | 45.5 | 39.1 | 50.93 | 48.9 | 58.53 | 60.2 | 54.03 |
| TIC (mg·L-1) | 23.251 | 22.702 | 22.378 | 20.084 | 17.426 | 24.769 | 26.094 | 29.16 | 27.454 | 22.672 |
| TC (mg·L-1) | 30.459 | 28.013 | 27.949 | 26.806 | 24.628 | 31.042 | 32.195 | 36.4 | 37.27 | 30.189 |
| TOC (mg·L-1) | 7.243 | 5.312 | 5.571 | 6.722 | 7.202 | 6.273 | 6.101 | 7.241 | 9.817 | 7.517 |
| TN (mg·L-1) | 1.474 | 1.281 | 0.976 | 0.785 | 0.897 | 0.779 | 0.998 | 0.892 | 1.244 | 0.876 |
| TP (mg·L-1) | 0.016 | 0.038 | 0.035 | 0.032 | 0.036 | 0.041 | 0.046 | 0.067 | 0.042 | 0.036 |
| ChlA (mg·L-1) | 0.122 | 0.079 | 0.065 | 0.042 | 0.081 | 0.121 | 0.119 | 0.079 | 0.056 | 0.131 |
|  | L3-3d | L3-7d | L3-15d | L3-30d | L3-60d | L4-3d | L4-7d | L4-15d | L4-30d | L4-60d |
| Latitude | 30.542 | 30.542 | 30.542 | 30.542 | 30.542 | 30.543 | 30.543 | 30.543 | 30.543 | 30.543 |
| Longitude | 114.424 | 114.424 | 114.424 | 114.424 | 114.424 | 114.415 | 114.415 | 114.415 | 114.415 | 114.415 |
| T | 23.9 | 22.8 | 25.2 | 25.3 | 29.2 | 22.4 | 22.6 | 24.7 | 25.1 | 28.7 |
| DO | 1.1 | 0.96 | 1.15 | 0.91 | 2.13 | 1.17 | 1.25 | 1.12 | 0.86 | 2.2 |
| C | 356.4 | 399.5 | 427.2 | 419 | 299 | 314.4 | 311 | 322.2 | 341.3 | 276.3 |
| pH | 7.73 | 7.31 | 8.09 | 7.65 | 8.00 | 7.62 | 7.63 | 8.07 | 7.58 | 7.89 |
| ORP | 88.9 | 57.8 | 82.2 | 108 | 99.2 | 95.7 | 93.5 | 72.6 | 110.2 | 100.9 |
| Turb | 31.77 | 35.07 | 39 | 38.8 | 37.83 | 35.5 | 39.43 | 37.8 | 53.83 | 40.43 |
| TIC (mg·L-1) | 23.633 | 29.167 | 27.111 | 28.329 | 3.43 | 21.9 | 22.07 | 21.776 | 23.395 | 20.234 |
| TC (mg·L-1) | 29.675 | 34.744 | 32.963 | 34.819 | 15.178 | 27.277 | 27.113 | 27.122 | 28.597 | 27.101 |
| TOC (mg·L-1) | 6.042 | 5.577 | 5.852 | 6.489 | 11.748 | 5.377 | 5.043 | 5.347 | 5.201 | 6.866 |
| TN (mg·L-1) | 1.055 | 1.109 | 1.041 | 1.056 | 2.387 | 1.215 | 1.46 | 1.095 | 1.427 | 2.038 |
| TP (mg·L-1) | 0.053 | 0.129 | 0.056 | 0.093 | 0.068 | 0.04 | 0.043 | 0.045 | 0.066 | 0.032 |
| ChlA (mg·L-1) | 0.115 | 0.046 | 0.148 | 0.083 | 0.043 | 0.101 | 0.106 | 0.123 | 0.057 | 0.082 |

**Note:** Dissolved oxygen (DO), temperature (T), pH, electrical conductivity (C), and oxidation-reduction potential (ORP) of the water were field measured by a portable multifunctional water quality analyzer (YSI CO., USA), and the turbidity (Turb) were measured using a portable turbidimeter instrument (Xinrui, China). The concentrations of total nitrogen (TN); total phosphorus (TP); total carbon (TC); total inorganic carbon (TIC); total organic carbon (TOC), and chlorophyll a (ChlA) in the water were determined according to previous studies [7, 8].

**Table S2.** Nested PERMANOVA performed to assess the effect of colonization time, colonizing environment, and polymer types on the composition of prokaryotic and eukaryotic microbial communities in the plastisphere. Asterisks indicate statistical significance (** *p*-value < 0.05 and *** *p*-value < 0.001). Meanwhile, the “Colonizing environment / Colonization time” indicates that the colonizing environment is nested on colonizing time.

| Factors | Prokaryotic microbiome | | Eukaryotic microbiome | |
| --- | --- | --- | --- | --- |
|  | R^2^ | *p*-value | R^2^ | *p*-value |
| **Colonizing environment / Colonization time** | **0.23318** | **0.0001***** | **0.28355** | **0.0001***** |
| **Colonization time / Colonizing environment** | **0.42742** | **0.0001***** | **0.40409** | **0.0001***** |
| Polymer type / Colonization time | 0.19649 | 0.0053** | 0.13394 | 1.0000 |
| Colonization time / Polymer type | 0.38706 | 0.0001*** | 0.29479 | 0.0001*** |
| Polymer type / Colonizing environment | 0.15029 | 0.9738 | 0.11229 | 1.0000 |
| Colonizing environment / Polymer type | 0.14661 | 0.5915 | 0.15259 | 0.5122 |

**Table S3.** PERMANOVA results showing differences between the different lakes of the free-living and particle-associated prokaryotic and eukaryotic microbiomes in the water. WF was the abbreviation of free-living in water, and WP was the abbreviation of particle-associated in water. Asterisks indicate statistical significance (* *p*-value < 0.05, ** *p*-value < 0.01, and *** *p*-value < 0.001).

| Factors | | Prokaryotic microbiome | | Eukaryotic microbiome | |
| --- | --- | --- | --- | --- | --- |
|  |  | R^2^ | *p*-value | R^2^ | *p*-value |
| WP | Colonization time | 0.29444 | 0.0048 ** | 0.28272 | 0.0199 * |
|  | Colonizing environment | 0.27882 | 0.0001 *** | 0.29732 | 0.0001 *** |
| WF | Colonization time | 0.32575 | 0.0171 * | 0.27597 | 0.0039 ** |
|  | Colonizing environment | 0.34756 | 0.0001 *** | 0.20583 | 0.0165 * |


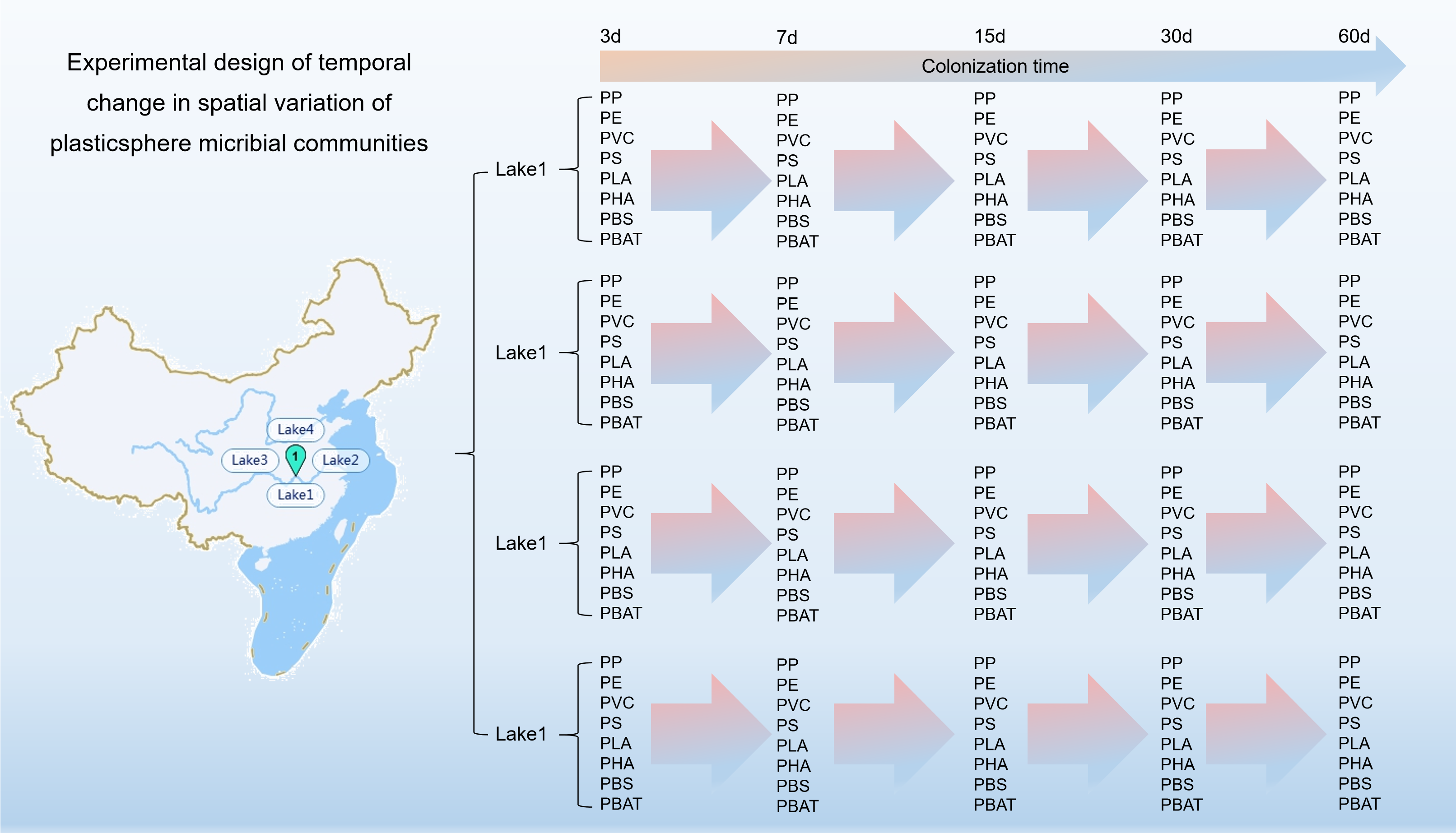


**Fig. S1**. Experimental design for determining temporal changes in spatial variation of plasticsphere prokaryotic communities in the freshwater lakes.


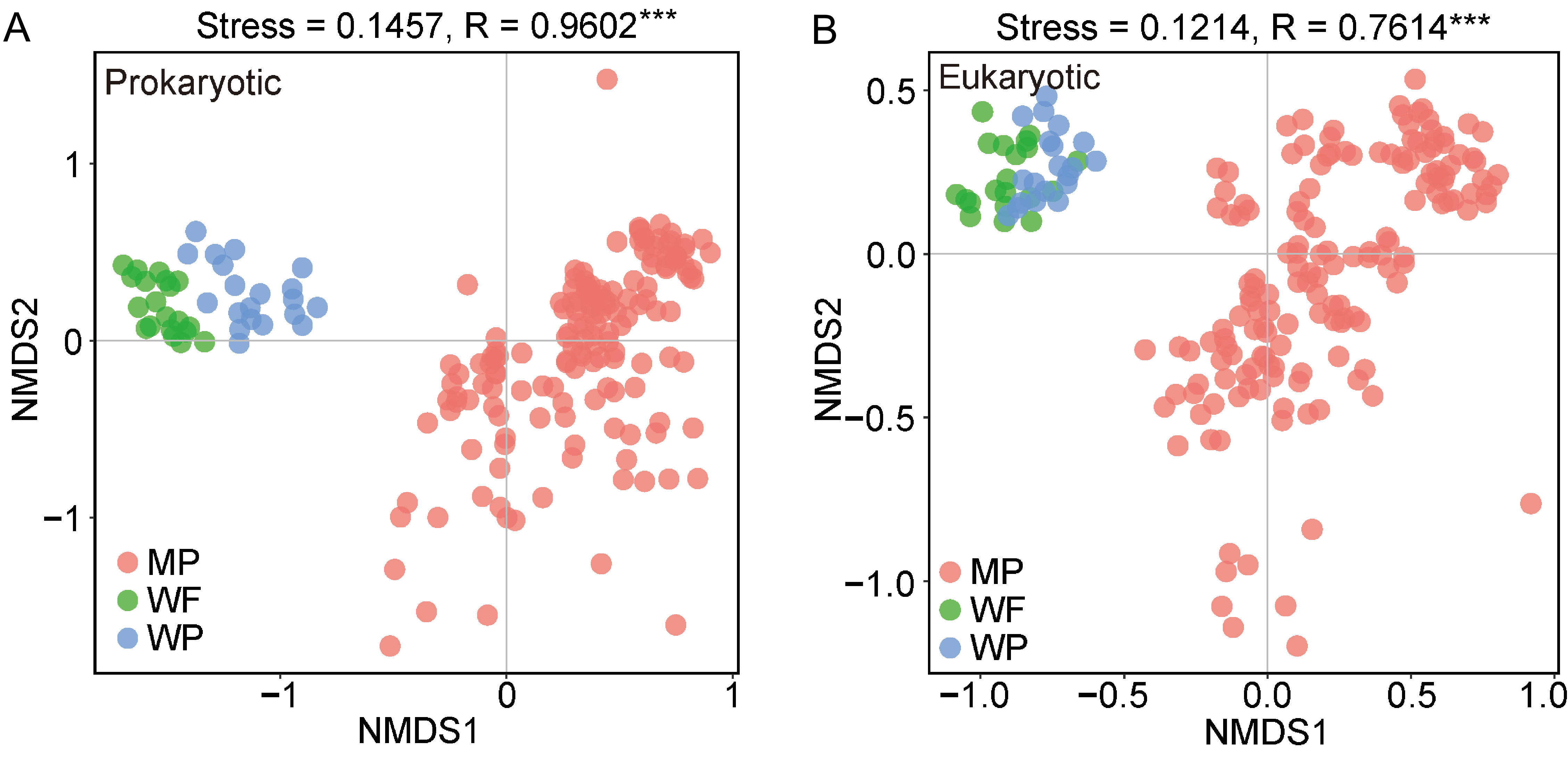


**Fig. S2.** NMDS results showed that the composition of plastisphere prokaryotic (A) and eukaryotic communities (B) was different from that of free-living and particle-associated prokaryotic and eukaryotic microbiomes in the water. In figure, MP was the abbreviation of plastisphere, WF was the abbreviation of free-living in water, and WP was the abbreviation of particle-associated in water. Asterisks indicate statistical significance (*** *p*-value < 0.001).


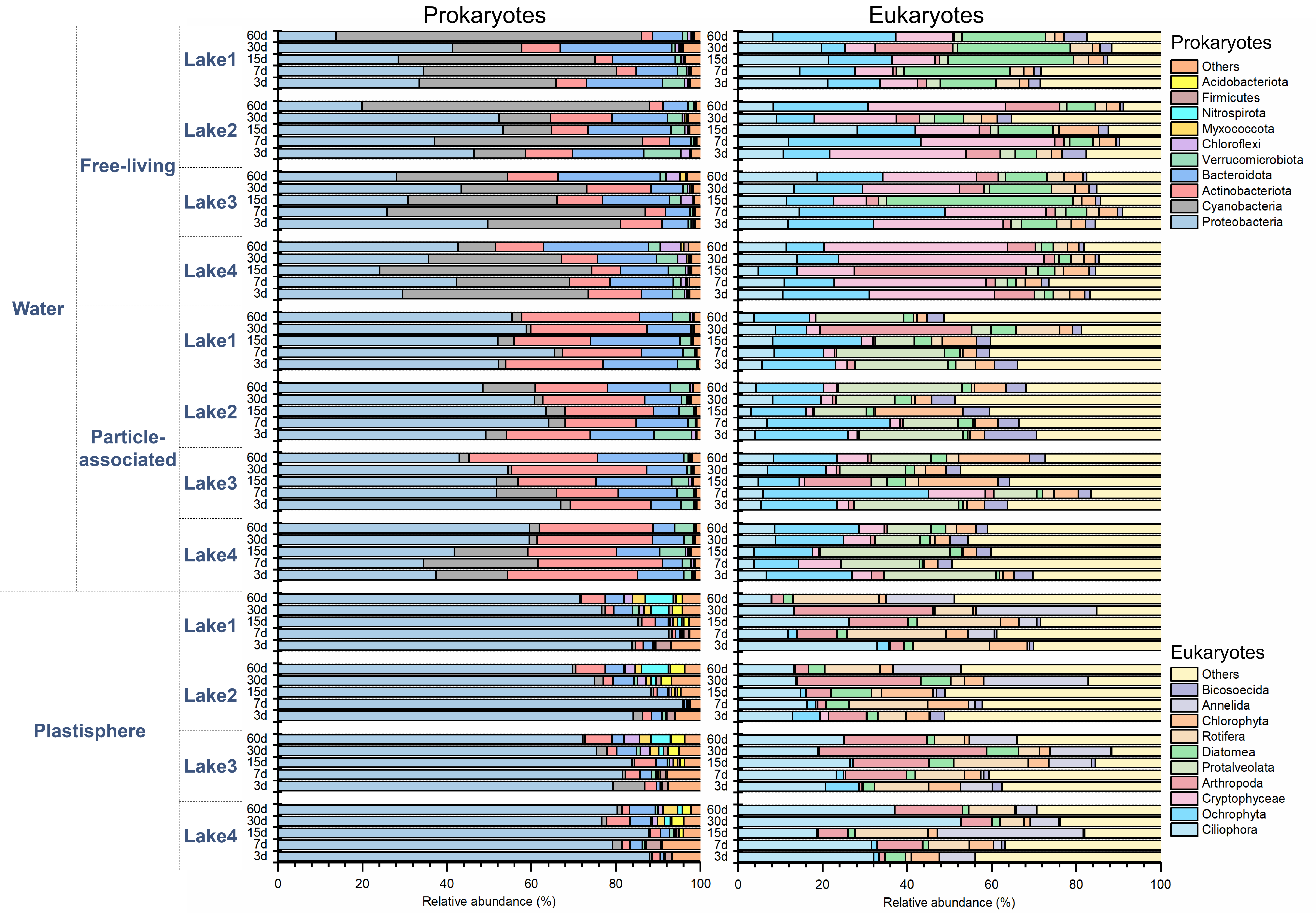


**Fig. S3.** The composition of prokaryotic and eukaryotic communities in the plastisphere and water at the phylum taxonomic level.


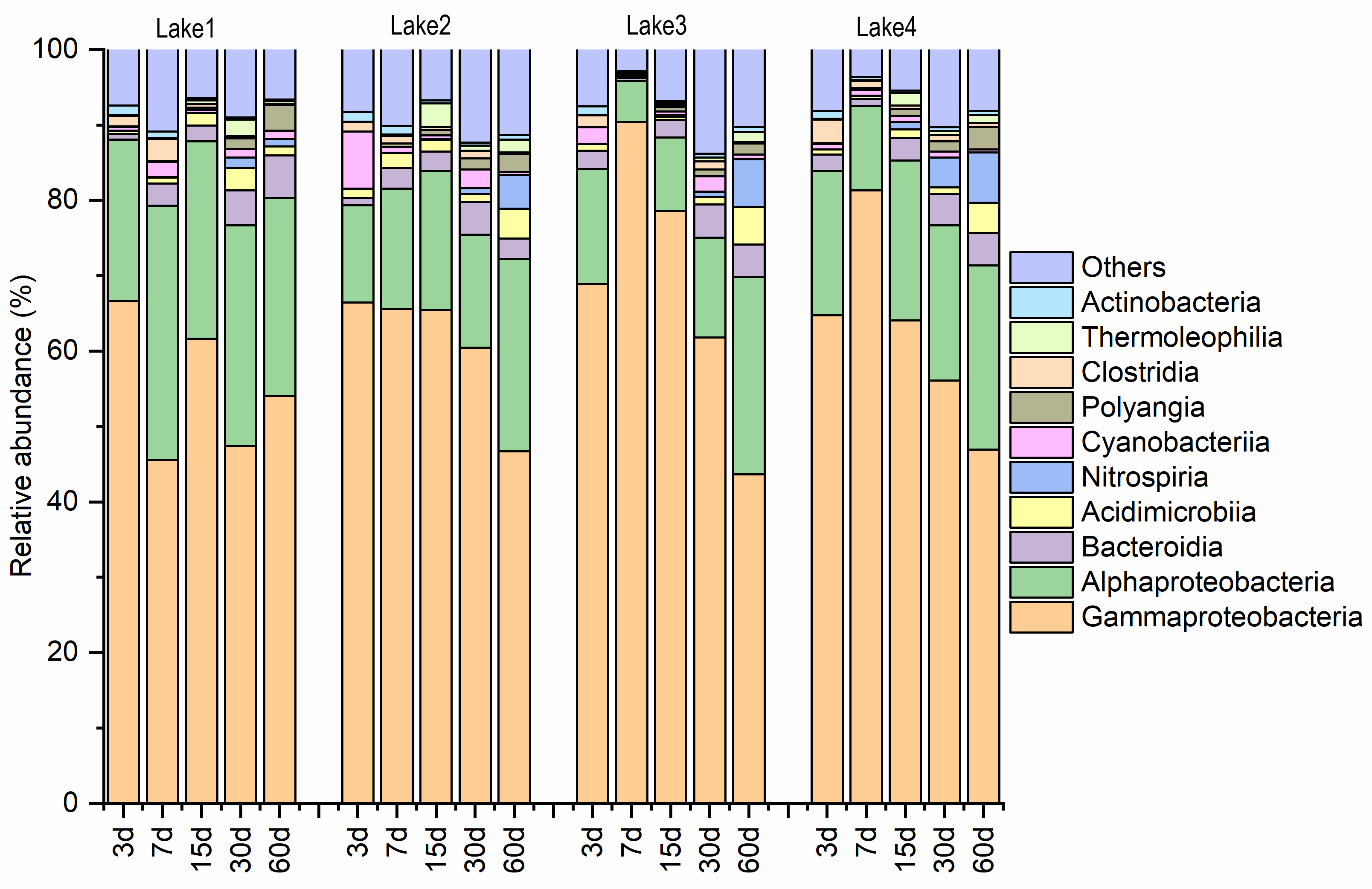


**Fig. S4.** The composition of prokaryotic communities in the plastisphere and water at the class taxonomic level.


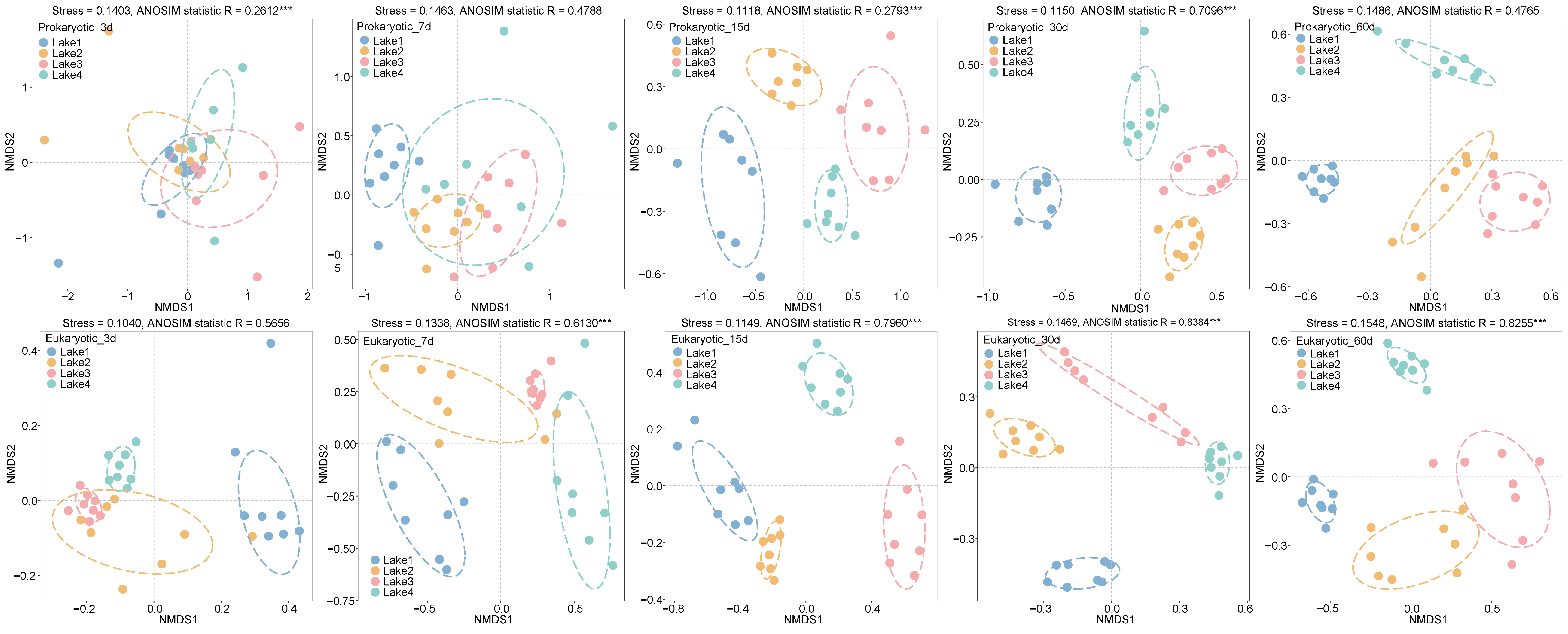
**Fig. S5.** NMDS results showed that the composition of plastisphere prokaryotic communities from different lakes were also different at the same colonization time. Similarly, the composition of plastisphere eukaryotic communities from different lakes were also different at the same colonization time. Asterisks indicate statistical significance (*** *p*-value < 0.001).


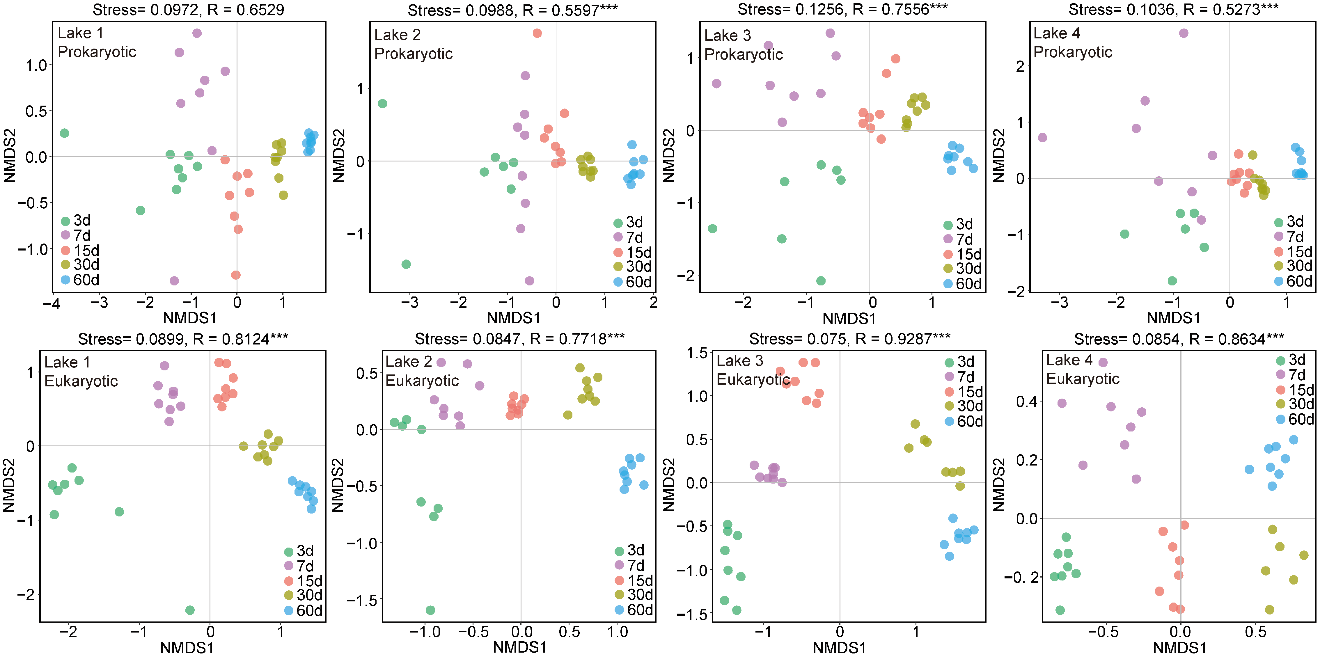


**Fig. S6.** Effects of colonization time on plastisphere microbial community composition in different lakes analyzed by NMDS. Asterisks indicate statistical significance (*** *p*-value < 0.001).


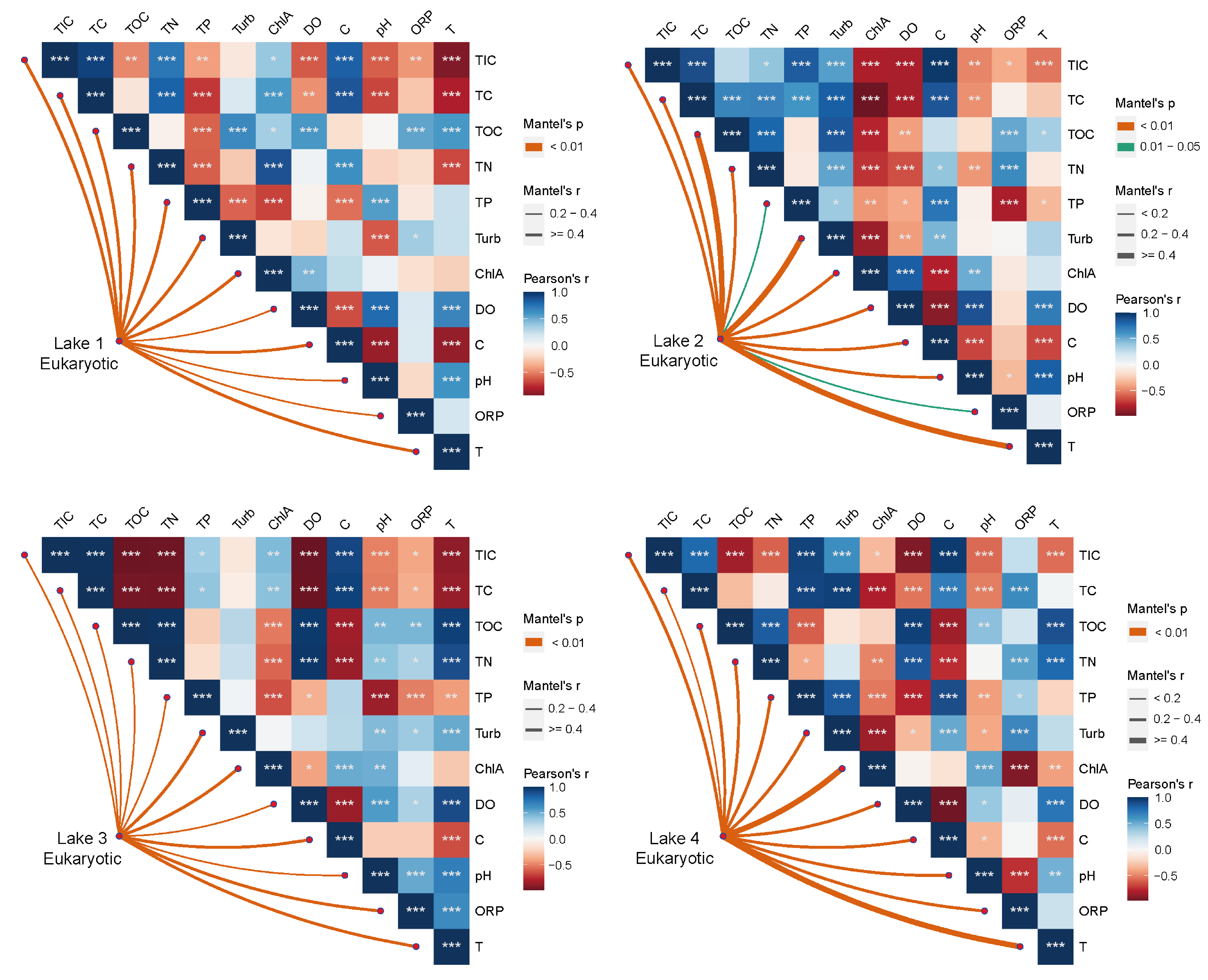


**Fig. S7.** Mantel test elucidation of the changes in the plastisphere eukaryotic communities composition in response to the surrounding water environment in the different lakes. TIC = total inorganic carbon, TC = total carbon, TOC = total organic carbon, TN = total nitrogen, TP = total phosphorus, Turb = Turbidity, ChlA = Chlorophyll a content, DO = dissolved oxygen, C = electrical conductivity, ORP = oxidation-reduction potential, and T=Temperature.

**

**

**Fig. S8**. Difference analysis of β_ratio_ between plastisphere prokaryotic and eukaryotic communities as well as between the different colonizing environments. β_ratio_ = β_sim_ / β_SOR_. Thus, β_ratio_ > 0.5 indicates that beta diversity was determined predominantly by species turnover, and β_ratio_ < 0.5 indicates nestedness was the dominant component. Asterisks indicate statistical significance (ns was *p*-value > 0.05, * *p*-value < 0.05, and *** *p*-value < 0.001). Different lowercase letters indicate that there are significant differences in the β_ratio_ of plastisphere prokaryotes between different lakes, and different uppercase letters indicate that there are significant differences in the β_ratio_ of plastisphere eukaryotes between different lakes (*p*-value < 0.05).





**Fig. S9**. The relative contributions of differentiating and homogenizing influences on the assembly of microbial communities in the plastisphere.

**References:**

1. Yang Y, Suyamud B, Liang S, Liang X, Wan W, Zhang W: Distinct spatiotemporal succession of bacterial generalists and specialists in the lacustrine plastisphere. *Environ. Microbiol*. 2023: 16400.

2. Eckert EM, Di Cesare A, Kettner MT, Arias-Andres M, Fontaneto D, Grossart H-P, Corno G: Microplastics increase impact of treated wastewater on freshwater microbial community. *Environ. Pollut.* 2018, 234:495-502.

3. Zhang M, Lu T, Paerl HW, Chen Y, Zhang Z, Zhou Z, et al. Feedback regulation between aquatic microorganisms and the bloom-forming *Cyanobacterium Microcystis* aeruginosa. *Appl. Environ. Microbiol.* 2019, 85(21): 01362-19.

4. Stegen JC, Lin X, Konopka AE, Fredrickson JK. Stochastic and deterministic assembly processes in subsurface microbial communities. *ISME J.* 2012, 6(9): 1653-64.

5. Kajan K, Osterholz H, Stegen J, Udovic MG, Orlic S. Mechanisms shaping dissolved organic matter and microbial community in lake ecosystems. *Water Res.* 2023, 245: 120653.

6. Zhang W, Bhagwat G, Palanisami T, Liang S, Wan W, Yang Y: Lacustrine plastisphere: Distinct succession and assembly processes of prokaryotic and eukaryotic communities and role of site, time, and polymer types. *Water Res.* 2024, 248: 120875..

7. Parker SP, Bowden WB, Flinn MB. The effect of acid strength and postacidification reaction time on the determination of chlorophyll a in ethanol extracts of aquatic periphyton. *Limnol. Oceanogr. Meth*. 2016, 14(12):839-852.

8. Zhang W, Suyamud B, Lohwacharin J, Yang Y. Large-scale pattern of resistance genes and bacterial community in the tap water along the middle and low reaches of the Yangtze River. *Ecotox. Environ. Safe.* 2021, 208: 111517.
